# Highly Efficient Protein Misfolding Cyclic Amplification

**DOI:** 10.1371/journal.ppat.1001277

**Published:** 2011-02-10

**Authors:** Nuria Gonzalez-Montalban, Natallia Makarava, Valeriy G. Ostapchenko, Regina Savtchenk, Irina Alexeeva, Robert G. Rohwer, Ilia V. Baskakov

**Affiliations:** 1 Center for Biomedical Engineering and Technology, University of Maryland, Baltimore, Maryland, United States of America; 2 Medical Research Service, Veterans Affairs Maryland Health Care System, Baltimore, Maryland, United States of America; 3 Department of Neurology, University of Maryland School of Medicine, Baltimore, Maryland, United States of America; 4 Department of Anatomy and Neurobiology, University of Maryland School of Medicine, Baltimore, Maryland, United States of America; University of Alberta, Canada

## Abstract

Protein misfolding cyclic amplification (PMCA) provides faithful replication of mammalian prions *in vitro* and has numerous applications in prion research. However, the low efficiency of conversion of PrP^C^ into PrP^Sc^ in PMCA limits the applicability of PMCA for many uses including structural studies of infectious prions. It also implies that only a small sub-fraction of PrP^C^ may be available for conversion. Here we show that the yield, rate, and robustness of prion conversion and the sensitivity of prion detection are significantly improved by a simple modification of the PMCA format. Conducting PMCA reactions in the presence of Teflon beads (PMCAb) increased the conversion of PrP^C^ into PrP^Sc^ from ∼10% to up to 100%. In PMCAb, a single 24-hour round consistently amplified PrP^Sc^ by 600-700-fold. Furthermore, the sensitivity of prion detection in one round (24 hours) increased by 2-3 orders of magnitude. Using serial PMCAb, a 10^12^-fold dilution of scrapie brain material could be amplified to the level detectible by Western blotting in 3 rounds (72 hours). The improvements in amplification efficiency were observed for the commonly used hamster 263K strain and for the synthetic strain SSLOW that otherwise amplifies poorly in PMCA. The increase in the amplification efficiency did not come at the expense of prion replication specificity. The current study demonstrates that poor conversion efficiencies observed previously have not been due to the scarcity of a sub-fraction of PrP^C^ susceptible to conversion nor due to limited concentrations of essential cellular cofactors required for conversion. The new PMCAb format offers immediate practical benefits and opens new avenues for developing fast ultrasensitive assays and for producing abundant quantities of PrP^Sc^ *in vitro*.

## Author Summary

Protein misfolding cyclic amplification (PMCA) provides faithful replication of mammalian prions *in vitro*. While PMCA has become an important tool in prion research, its application is limited because of low yield, poor efficiency and, sometimes, stochastic behavior. The current study introduces a new PMCA format that dramatically improves the efficiency, yield, and robustness of prion conversion *in vitro* and reduces the time of the reaction. These improvements have numerous implications. The method opens new opportunities for improving prion detection and for generating large amounts of PrP^Sc^ *in vitro*. Furthermore, the results demonstrate that *in vitro* conversion is not limited by lack of convertible PrP^C^ nor by concentrations of cellular cofactors required for prion conversion.

## Introduction

Protein misfolding cyclic amplification (PMCA)^2^ provides faithful amplification of mammalian prions *in vitro* and, since its introduction in 2001 [1], has become an important tool in prion research. To date, PMCA provides the most sensitive approach for detecting miniscule amounts of prion infectivity [2]–[5], including detection of prions in blood or peripheral tissues at preclinical stages of the disease [6]–[8]. In recent studies, PMCA was employed for generating infectious prions (PrP^Sc^) *in vitro de novo* in crude brain homogenate [9], and for producing infections prions from the cellular prion protein (PrP^C^) purified from normal mammalian brains [10] and recombinant PrP (rPrP) produced in *E. coli* [11]. Furthermore, PMCA has been used for identifying cofactors that are involved in prion replication [12]–[15] and assessing the impact of glycosylation on replication of prion strains [16]. PMCA has also been utilized for assessing the prion transmission barrier [17], [18], prion interference [5] and adaptation to new hosts [19].

PMCA reactions consist of two alternating steps: incubation and sonication. Sonication fragments PrP^Sc^ particles or fibrils into smaller pieces, a process that that is believed to result in the multiplication of active centers of PrP^Sc^ growth. During the incubation step, small PrP^Sc^ particles grow by recruiting and converting PrP^C^ molecules into PrP^Sc^.

While the discovery of PMCA has provided new opportunities for exploring the prion replication mechanism, the low yield of PrP^Sc^ has limited its utility for structural studies. Furthermore, the efficiency of amplification in PMCA varies dramatically depending on minor variations in experimental parameters, including those that are difficult to control, such as the age of the sonicator's horn and individual patterns of horn corrosion. Previous strategies for improving the efficiency of PMCA focused on increasing the number of cycles within a single PMCA round [2] or increasing the substrate concentration by using a normal brain homogenate (NBH) from transgenic mice overexpressing PrP^C^ [20], [21]. Here we describe a new PMCA format that employs beads (referred to as PMCAb). Supplementing the reaction with beads resulted in remarkable improvements in the yield, rate and robustness of prion conversion, as well as in the sensitivity of prion detection. This simple modification of the PMCA format enables fast and efficient production of high quantities of PrP^Sc^. This result also shows that the low yield observed previously has not been due to a lack of PrP^C^ susceptible to conversion, nor has it been limited by cellular cofactors.

## Results

### Beads significantly improve the yield and rate of PMCA reactions

**Figure 1:**
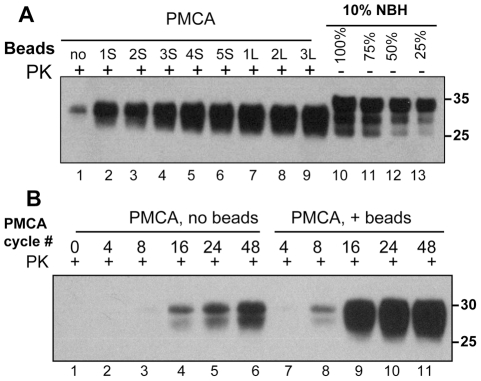
Beads improve the yield and rate of PrP^Sc^ conversion. (**A**) 263K scrapie brain material was diluted 10^3^-fold into 10% normal brain homogenate (NBH) and subjected to 48 PMCA cycles in the absence of beads (lane 1) or presence of 1, 2, 3, 4 or 5 small beads (S, lanes 2–6) or 1, 2 or 3 large beads (L, lanes 7–9). 10% NBH loaded at 100%, 75%, 50% or 25% amounts were used to estimate the amplification yield (lanes 10–13, respectively). (**B**) 263K scrapie brain material was diluted 10^3^-fold into 10% NBH and subjected to 4, 8, 16, 24 or 48 PMCA cycles in the absence of beads (lanes 2–6) or presence of 5 small beads (lanes 7–11). Prior to electrophoresis, samples were digested with Proteinase K (PK) as indicated.

In the past, we found that beads with a diameters of 1.59 mm (referred to as small or S) or 2.38 mm (referred to as large or L) significantly accelerated the formation of amyloid fibrils of rPrP *in vitro* [22]. Here, we tested whether beads have any effects on the rate of prion amplification in PMCA. In standard PMCA (sonication for 30 sec every 30 min, 48 cycles total, no beads), the typical yield of conversion of PrP^C^ into PrP^Sc^ was approximately 10% as judged by Western blotting (Fig. 1A). This amplification yield was consistent with previous studies on amplification of the 263K strain. In the presence of beads, however, the conversion yield improved significantly and approached 100% when 3 large or 5 small beads were used (Fig. 1A).

The kinetics of PrP^Sc^ amplification monitored by Western blotting revealed that in the absence of beads the newly generated PrP^Sc^ was detected by the 16^th^ cycle, whereas in the presence of beads it already was seen by the 8^th^ cycle (Fig. 1B). Furthermore, in the presence of beads the reaction reached a plateau in only 24 cycles and produced a much higher yield (Fig. 1B). These results illustrated that beads with diameters of 1.59 or 2.38 mm improved both the yield and the rate of 263K conversion. When beads of submillimeter diameter (800, 400, 200 or 100 µm, see Methods) were used instead, no noticeable increase in PrP^Sc^ amplification was observed (data not shown).

### PMCAb amplifies prion infectivity

**Figure 2:**
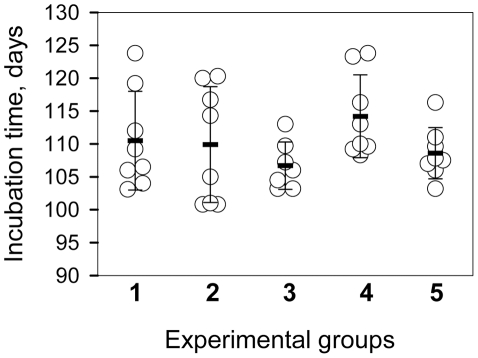
Bioassay of PMCA products. Incubation time to terminal disease stage in individual animals (circles) and in animal groups represented as mean ± S.D. (black bars). Animals were inoculated with 10^−4^-fold diluted 263K brain homogenate (group 1); 10^−4^-fold diluted 263K brain homogenate sonicated for 48 cycles (a single PMCA round) in the absence of beads (group 2) or presence of 3 large beads (group 3) in the absence of NBH; and 10^−4^-diluted 263K brain homogenate subjected to six serial PMCA rounds in the absence of beads (group 4) or presence of 3 large beads (group 5) using 1∶10 dilutions between rounds. After serial PMCA, the amplification products were diluted an additional 10-fold to obtain 10^10^-fold dilutions of the original seeds for inoculating groups 4 and 5.

To test whether the products of PMCAb were infectious, the reactions were seeded with 10^4^-diluted 263K brain material and subjected to amplification in the presence or absence of beads for 6 rounds of 48 cycles each. The products of each round were diluted 10-fold into fresh NBH for the subsequent round. The PMCA products from the final round were then diluted an additional 10-fold prior to inoculation of 50 µl per animal. The final dilution of the initial 263K brain material was 10^10^ fold. In our laboratory the concentration of 263K scrapie in the brains of hamsters in the late stages of symptomatic disease is consistently between 1 and 2×10^10^ Infectious Dose_50_/g of brain [23]. In the absence of amplification, a 10^10^ dilution of 263K brain would contain 1 ID_50_/ml giving a probability of infection of 0.05 from a 50 µl inoculation [23]. Nevertheless, all animals inoculated with PMCA products formed in the presence or absence of beads developed clinical disease with the mean value of endpoint 108.6±3.9 or 114.2±6.3 days post inoculation, respectively (Fig. 2, groups 4 and 5, respectively). Incubation time endpoints were determined empirically as described in the methods and [23]. The reference group inoculated with 10^4^-diluted 263K brain reached the endpoint by 110.5±7.5 days (Fig. 2, group 1). Bioassays of two 263K brain homogenates sonicated for 48 PMCA cycles (1 round) in the absence of a substrate revealed that sonication of PrP^Sc^ per se did not notably change its infectivity level regardless of whether beads were present or absent during the sonication cycles (Fig. 2, groups 2 and 3, respectively). Similar amounts of PrP^Sc^ were found in the brains from all animal groups (Fig. S1).

The bioassay experiment confirmed that prion infectivity is amplified in PMCAb. Without a titration experiment, it is difficult to establish accurate infectivity titers of PMCA or PMCAb products. Nevertheless, considering that group 5 gave the same incubation times as group 1, even though the amplification products were diluted an additional 10-fold prior to inoculation, the infectivity dose of PMCAb products appeared to be 10-fold higher than the dose in 10^4^-diluted 263K brain material.

### The sensitivity of PrP^Sc^ detection is improved in PMCAb

**Figure 3:**
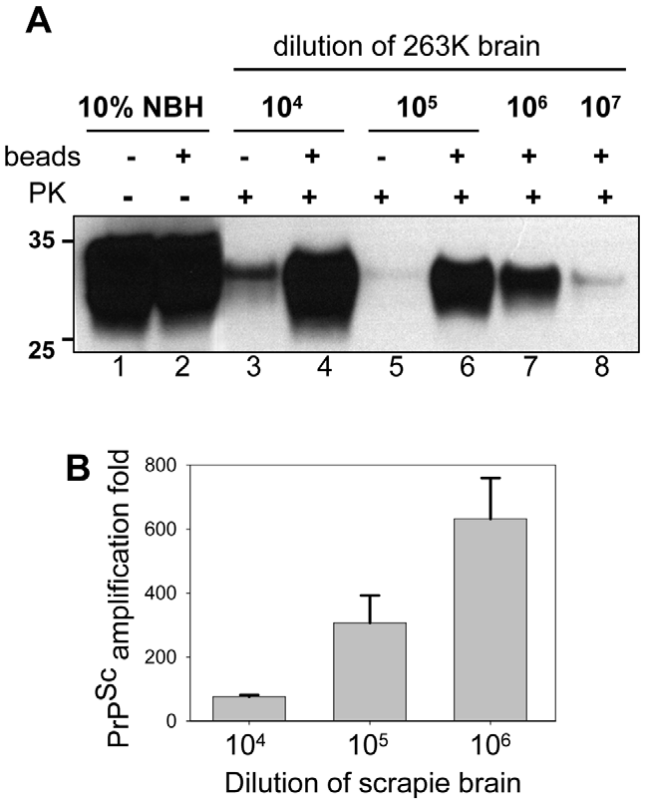
Beads improve the sensitivity of PrP^Sc^ detection. (**A**) 263K scrapie brain material was serially diluted 10^4^-, 10^5^-, 10^6^- or 10^7^- fold into 10% NBH, subjected to 48 PMCA cycles in the absence of beads (lanes 3,5) or presence of five small beads (lanes 4,6,7,8) and digested with PK. Undigested 10% NBH (lanes 1,2) was loaded as a reference. (**B**) PrP^Sc^ amplification in 48 cycles of PMCA conducted with five small beads for 263K scrapie brain material serially diluted 10^4^-, 10^5^- or 10^6^-folds as analyzed by dot blotting. The data represent average and standard deviation of three independent PMCA reactions. The amplification fold was calculated using calibration curve in Fig. S3.

To test whether the application of beads improves the detection limit, serially diluted 263K brain homogenate was used to seed the PMCA reactions that consisted of 48 cycles. In the absence of beads, seeding with 10^3^-fold and with 10^4^-fold diluted scrapie brains gave sufficient amplification of PrP^Sc^ to be detected by Western blotting. In the presence of beads, however, the reactions seeded with 10^6^-fold diluted 263K brains showed consistent, reproducible amplification for subsequent detection by Western blotting (Fig. 3). Frequently, sufficient amplification of PrP^Sc^ for detection by Western blotting was observed in the reactions with beads seeded with 10^7^-fold diluted scrapie brains. Therefore, within 48-cycle PMCA, beads improved the sensitivity of detection by at least 2 or 3 orders of magnitude. To rule out the possibility of the PrP^Sc^ formation *de novo*, 32 unseeded reactions were conducted, each of which consistent of 3 rounds of serial PMCAb (sPMCAb). None of them showed PK-resistant material on Western blotting (Fig. S2).

To estimate quantitatively the PrP^Sc^ amplification fold achieved in a single PMCAb round, we employed dot blotting as it provides a better linear response within a broader range of PrP^Sc^ concentrations than the Western blotting (Fig. S3). The PMCAb reactions seeded with 10^4^-, 10^5^-, or 10^6^-diluted 263K brain were found to produce reliable amplification by ∼75-, 300-, and 635- fold within 48 cycles, respectively (Fig. 3B). An increase in amplification fold at higher dilutions of seeds suggests that the effect of beads was most beneficial at high PrP^C^ to PrP^Sc^ ratios, where the reaction is no longer limited by the concentration of a substrate and/or cofactors.

To estimate the PrP^Sc^ amplification fold using an alternative approach, sPMCA reactions consisted of three rounds were performed with the dilution factors between the rounds ranging from 1∶10 to 1∶1000. In the absence of beads, we observed a gradual decrease in the signal intensity as a function of PMCA round at dilutions of 1∶20 indicating that the amplification fold in each round was slightly lower than 20 (Fig. S4). In the presence of beads, however, the signal was stable at 1∶100 dilution but decayed at 1∶1000 dilution, suggesting that the amplification fold in each round was higher than 100 but less than 1000 (Fig. S4). This experiment confirmed that up to several hundred fold amplification could be achieved in one PMCAb round consisted of 48 cycles, if the reaction is not limited by substrate and cofactors.

### Detection of minute amounts of PrP^Sc^

**Figure 4:**
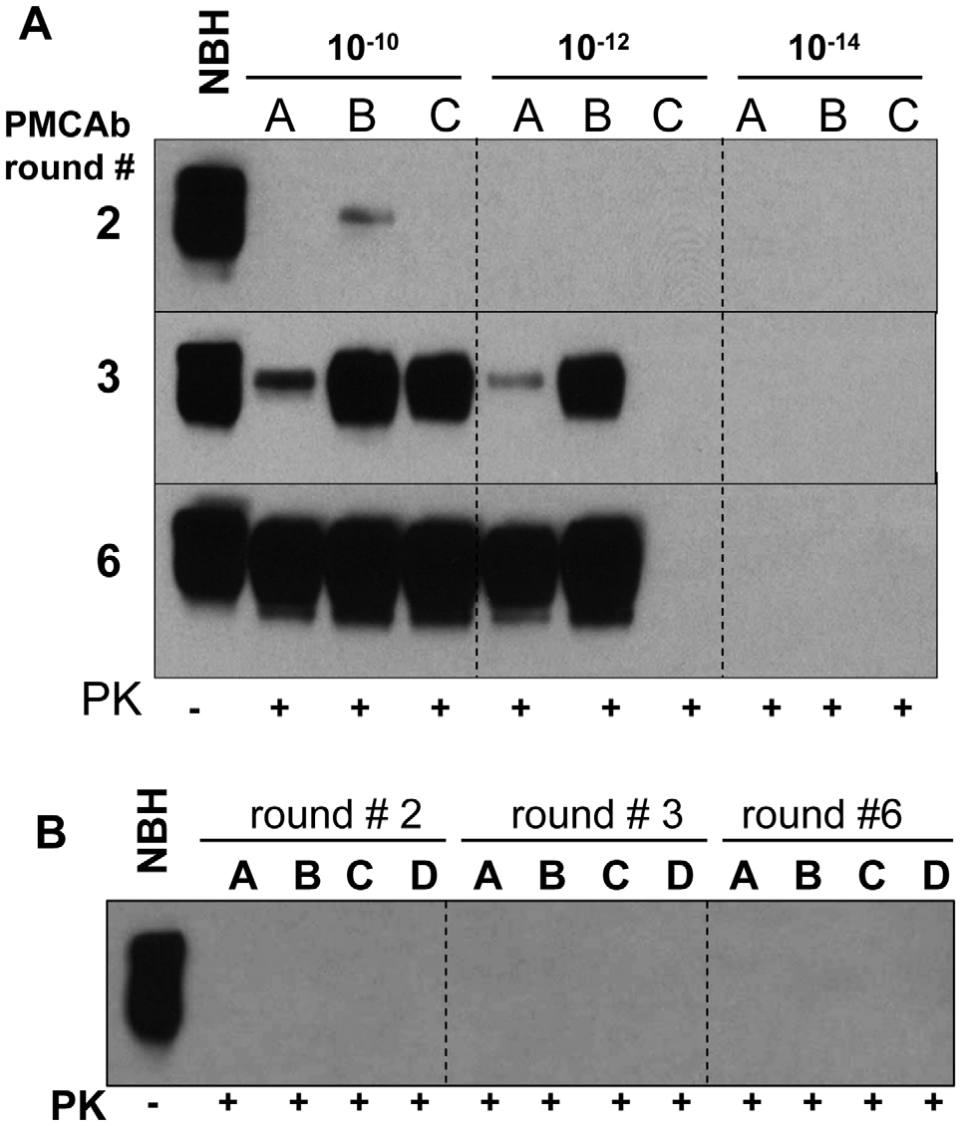
Amplification of minute amounts of PrP^Sc^ in PMCAb. (**A**) 263K brain material was serially diluted 10^10^-, 10^12^-, or 10^14^-fold and subjected to sPMCAb amplification in the presence of 3 large beads for 6 rounds as indicated (each round consists of 48 sonication cycles, 10-fold dilutions were used for subsequent rounds). Amplification in three independent experiments (A, B, and C) are shown. (**B**) Up to six rounds of PMCAb in non-seeded NBHs (reactions A and B) or in NBHs seeded with 10 µl of 10% NBH prepared from 661 days old Syrian Hamsters (reactions C and D) were performed as negative controls. Undigested 10% NBH is provided as a reference.

In previous studies, sPMCA of serially diluted 263K brain homogenate was used to determine the last dilution that still contained PrP^Sc^ particles [2]. Three out of four reactions seeded with 10^12^-diluted 263K brain material were found to be positive, while five to seven sPMCA rounds, each consisting of 144 cycles, were required to amplify 10^12^-diluted 263K to levels detectible by Western blotting [2]. To test the effectiveness of PMCAb in amplifying minute quantities of PrP^Sc^, 263K brain homogenate was serially diluted up to 10^14^-fold and then amplified in sPMCAb, where each round consisted of 48 cycles. 10^12^-diluted 263K brain material was detected in 4 out of 8 reactions in the third round (Fig. 4A). An increase in number of rounds to six did not increase the percentile of positive reactions seeded with 10^12^-diluted 263K brain nor did it reveal any positive signals in reactions seeded with 10^14^-diluted 263K brain (Fig. 4A). 10^10^-diluted 263K brains showed a positive signal in all independent reactions (Fig. 4A). Non-seeded reactions or reactions seeded with NBH from old animals showed no positive signals in PMCAb (Fig. 4B). These results are consistent with the previous studies where brain material diluted 10^12^-fold detected PrP^Sc^ and showed stochastic behavior [2] consistent with a limiting dilution of the signal [24]. In the current experiments, PMCAb achieved the same level of sensitivity as PMCA in 1/7^th^ of the time and with no evidence of spontaneous conversion from NHB substrate.

### Beads improve the amplification rate of a synthetic prion strain

**Figure 5:**
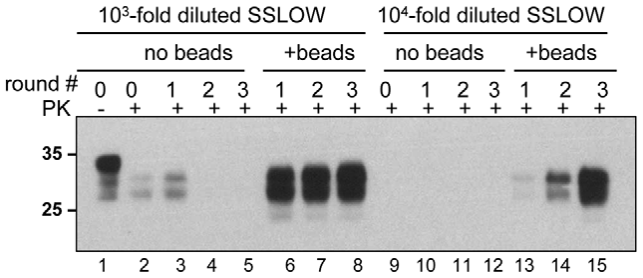
Beads improve the amplification efficiency of SSLOW. SSLOW scrapie brain material was diluted 10^3^-fold (lanes 1–8) or 10^4^-fold (lanes 9–15) into 10% NBH, subjected to serial PMCA in the absence or presence of 3 small beads, as indicated, and digested with PK. Each PMCA round consisted of 48 cycles; the material amplified in each round was diluted 10-fold into 10% NBH for the next PMCA round. Undigested 10% NBH (lane 1) loaded at 1/10^th^ the amount of the digested samples is provided as a reference.

To test whether the positive effect of beads on prion amplification was limited to 263K, we used a synthetic prion strain, SSLOW, which was previously found to have a very peculiar amplification behavior in PMCA [25]. Previously we found that amplification efficiency of SSLOW varied significantly from preparation to preparation of NBH and that it had a much more unstable amplification behavior than 263K. For instance, SSLOW failed to amplify even in those preparations of NBHs, where 263K showed high amplification rates. In such preparations of NBHs, the amplification fold for SSLOW was found to be lower than the 10-fold dilution factor used for serial PMCA. Therefore, in the absence of beads, SSLOW PrP^Sc^ was no longer detectable by Western blotting after the first round of PMCA (Fig. 5, *lanes 3–5*). In the presence of beads, however, the amount of SSLOW PrP^Sc^ remained stable during serial PMCAb if the reactions were seeded with 10^3^-fold diluted SSLOW brain homogenates (Fig. 5, *lanes 6–8*), or increased if 10^4^-fold dilutions were used for seeding (Fig. 5, *lanes 13-15*). These results illustrate that the positive effect of beads is not limited to 263K and that beads improved the robustness of PMCA for a strain with poor amplification behavior.

### Beads counteract the negative effect of recombinant PrP

**Figure 6:**
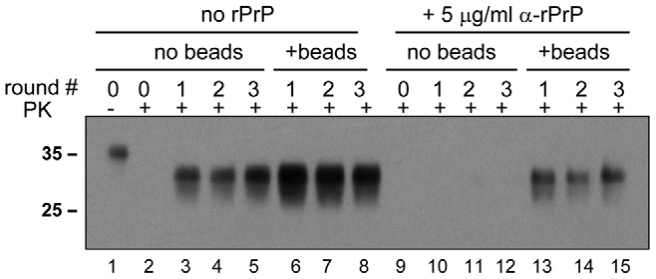
Beads counteract the negative effect of rPrP on PrP^Sc^ amplification. 263K scrapie brain material was diluted 10^3^-fold into 10% NBH and subjected to a serial PMCA in the absence or presence of α-rPrP (5 µg/ml) and absence or presence of 3 small beads, as indicated. Each PMCA round consisted of 48 cycles; the material amplified in each round was diluted 10-fold into 10% NBH for the next PMCA round. Undigested 10% NBH (lane 1) loaded at 1/10^th^ the amount of the digested samples is provided as a reference.

In previous studies, recombinant PrP (rPrP) was found to inhibit amplification of PrP^Sc^ in PMCA [16]. To test whether the inhibitory effect can be rescued by addition of beads, serial PMCA was performed in the absence or presence of 5 µg/ml Syrian hamster full-length rPrP folded into a α-helical conformation. In the absence of beads, rPrP was found to suppress the amplification of 263K (Fig. 6, *lanes 10–12*). The addition of beads, however, restored the amplification rate of 263K to the level observed in the absence of rPrP and beads (Fig. 6, compare *lanes 13–15* to *3–5*). However, this amplification level was lower than those observed in the presence of beads without rPrP (Fig. 6, *lanes 6–8*).

### Species-specificity is preserved in PMCAb

**Figure 7:**
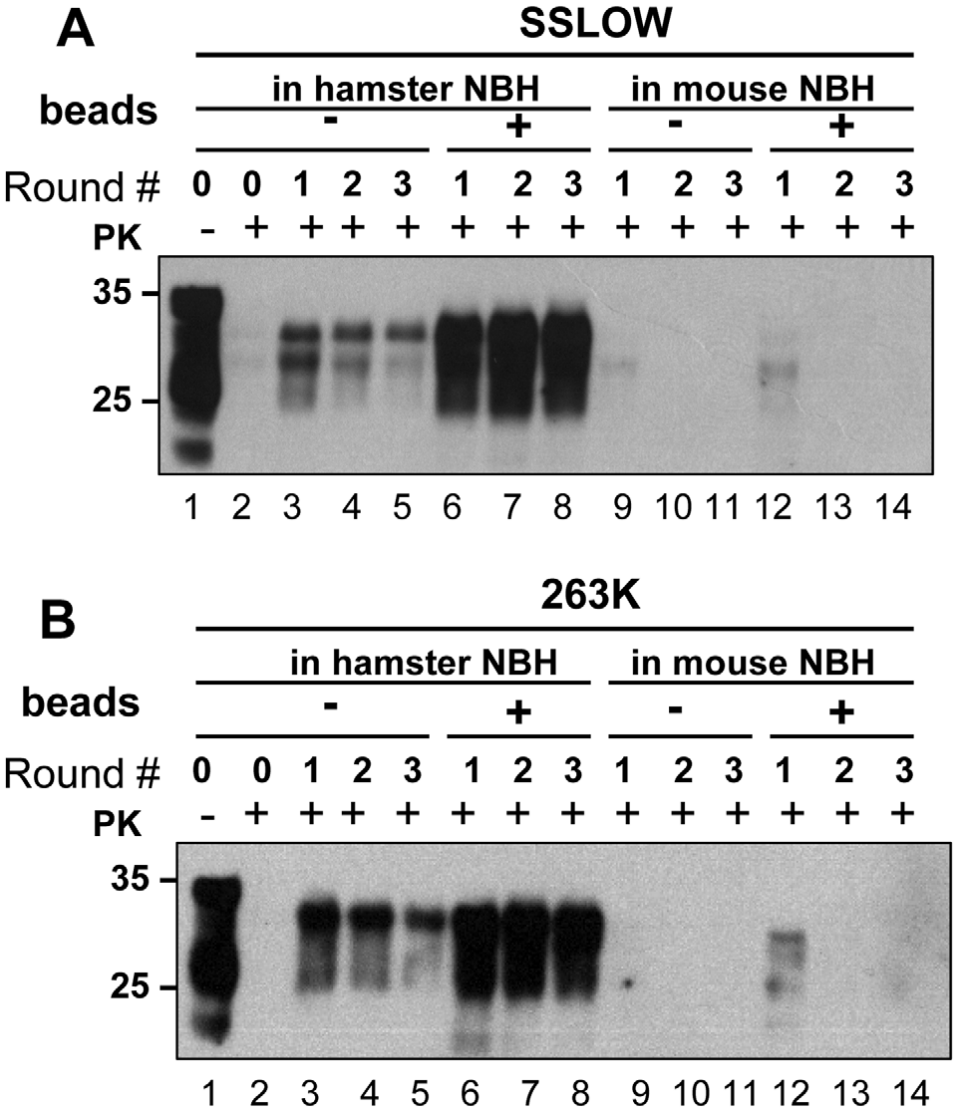
PMCA with beads preserves species barrier. (**A**) SSLOW scrapie brain material was diluted 10^3^-fold into 10% hamster or 10% mouse NBH, subjected to serial PMCA in the absence or presence of 3 large beads, as indicated, and digested with PK. Undigested 10% NBH (lane 1) is provided as a reference. (**B**) 263K scrapie brain material was diluted 10^3^-fold into 10% hamster or 10% mouse NBH, subjected to serial PMCA in the absence or presence of 3 large beads, as indicated, and digested with PK. Undigested 10% NBH (lane 1) is provided as a reference. Each PMCA round consisted of 48 cycles; the material amplified in each round was diluted 10-fold into 10% NBH for the next PMCA round.

**Figure 8:**
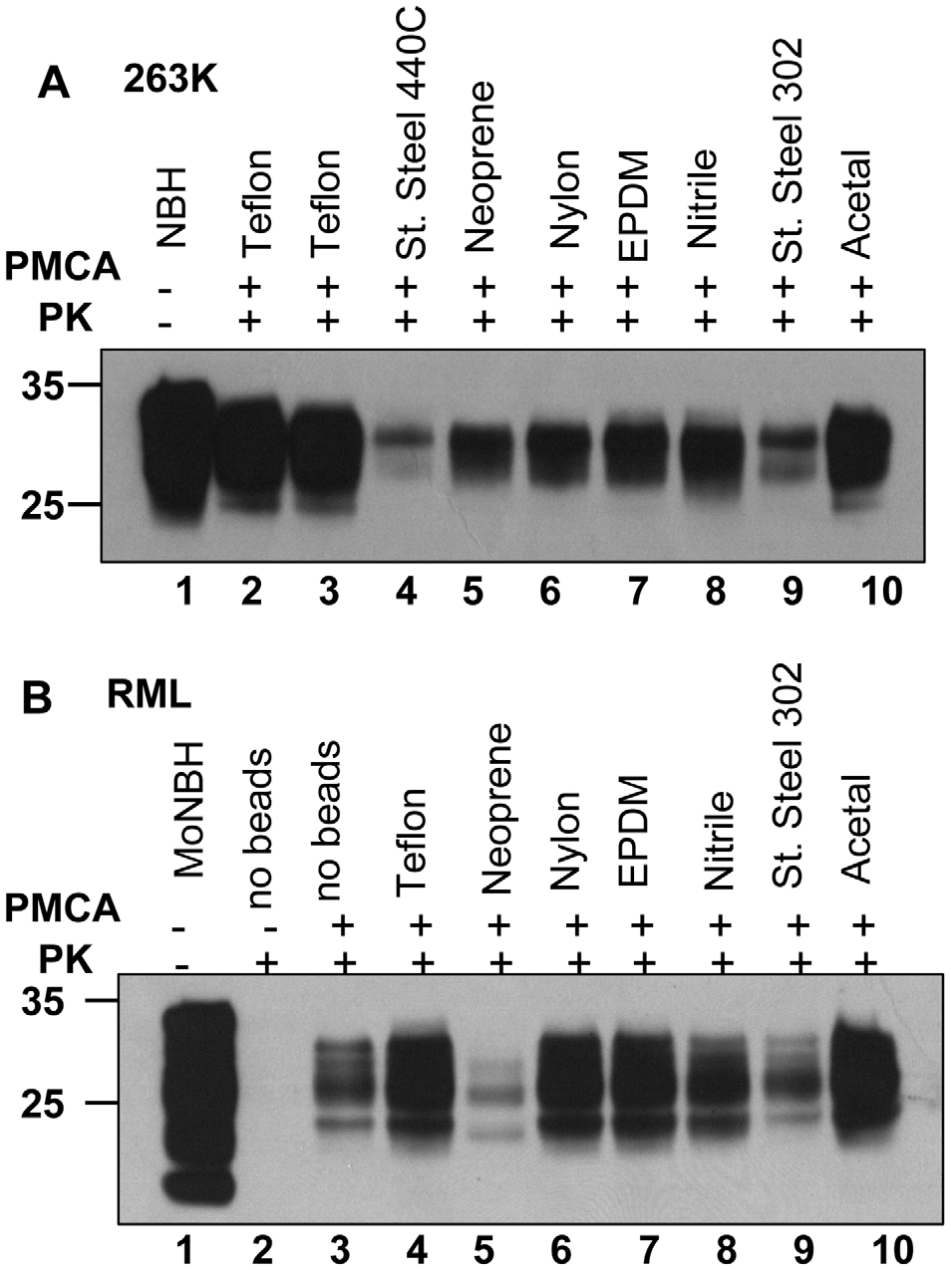
Effect of bead material on efficiency of amplification. (**A**) 263K scrapie brain material was serially diluted 10^5^-fold into 10% hamster NBH and subjected to 48 PMCA cycles in the presence of two beads made from Teflon (purchase from McMaster-Carr – lane 2, or Small Parts – lane 3), stainless steel 440C, neoprene, nylon, EPDM, nitrile, stainless still 302, or acetal, as indicated. Prior to electrophoresis, samples in lanes 2–10 were digested with PK. Undigested 10% hamster NBH (lane 1) was loaded as a reference. Without PMCA, 10^5^- fold diluted 263K brain material was not detectable by Western blotting (not shown). (**B**) RML scrapie brain material was serially diluted 10^4^-fold into 10% mouse NBH and subjected to 48 PMCA cycles in the absence of beads (lane 3) or presence of two beads made from Teflon (purchase from Small Parts – lane 4), neoprene, nylon, EPDM, nitrile, stainless still 302, or acetal, as indicated. Prior to electrophoresis, samples in lanes 2–10 were digested with PK. Undigested 10% mouse NBH (lane 1) was loaded as a reference.

Prion amplification in PMCA was previously shown to exhibit species specificity that faithfully reflects the transmission barrier observed in animals [17], [18]. Considering that beads were found to improve significantly the amplification efficiency, we were interested in testing whether the species specificity was preserved in PMCAb. To address this question, two hamster strains, 263K and SSLOW were used to seed PMCA reactions in mouse NBHs. Consistent with the previous results, beads improved the conversion yield for both strains when they were amplified in Syrian hamster NBH (Fig. 7A,B). However, when 263K or SSLOW were diluted with mouse NBH no detectible amplification was observed for at least three serial PMCA rounds in the presence or absence of beads (Fig. 7A,B). A control experiment revealed that mouse RML strain could be amplified in mouse NBH (data not shown, and Fig. 8B). Therefore, the lack of detectible amplification of hamster strains in serial PMCA in mouse NBH confirmed that the presence of beads does not eliminate the species barrier. Taken together, these results illustrate that significant improvements in amplification efficiency do not come at the expense of amplification specificity.

### Effect of bead material on efficiency of amplification

To test whether efficiency of amplification depends on the bead material, beads made from eight different materials including Teflon beads purchased from two companies were used for amplification of 10^5^- fold diluted 263K or 10^4^- fold diluted RML (Fig. 8A,B). Beads made from Teflon and acetal showed the best amplification efficiency for both strains. Nylon and EPDM beads showed very good performance in amplifying RML, but were less efficient for 263K. Notably, the ranking orders in amplification efficiency for different materials appeared to be strain- or species-dependent. The detailed relationship between the bead material and their efficiencies to amplify different scrapie strains or strains from different species will be explored in future studies.

### Sonication with beads fragments amyloid fibrils of rPrP into small pieces

**Figure 9:**
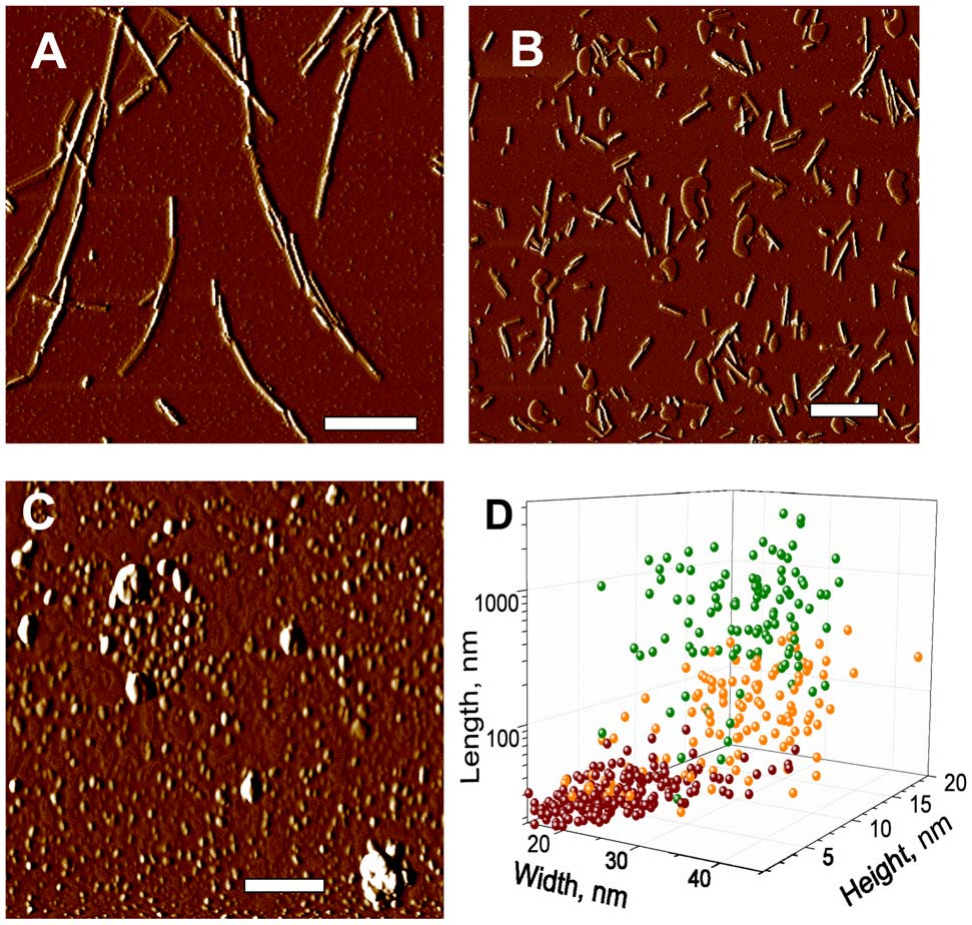
AFM imaging of rPrP fibril fragmentation. AFM imaging of intact rPrP fibrils (**A**) and fibrils sonicated for 30 sec in the absence (**B**) or presence of 5 small beads (**C**) using sonication conditions identical to those used in PMCA. Scale bars  = 0.5 µm. (**D**) Analysis of length, width and height for intact rPrP fibrils (*green circles*) and fibrils sonicated in the absence (*orange circles*) or presence of 5 small beads (*red circles)*.

To gain insight into the effect of beads on prion amplification, we tested whether beads affect the fragmentation efficiency of PrP aggregates during sonication. Amyloid fibrils produced from rPrP were sonicated in the presence or absence of beads, and the size of fibrillar fragments was analyzed using atomic force microscopy (AFM) imaging. Consistent with our previous studies [26], sonication was found to break fibrils into smaller fragments (Fig. 9A,B). Sonication in the presence of beads, however, reduced the size of fibrillar fragments even more producing smaller particles (Fig. 9C,D). In fact, AFM imaging revealed that after sonication with beads, the fibrillar fragments appeared as small oligomers.

## Discussion

The current studies demonstrated that the yield and the rate of prion conversion in PMCA can be substantially improved by including beads. Remarkably, substantial improvements in the amplification efficiency and robustness did not come at the cost of prion replication specificity. While beads were found to increase the amplification rate of two hamster strains in hamster NBHs, no detectible amplification of these strains were observed in mouse NBHs within three rounds. This shows that the species specificity was preserved (Fig. 7). Furthermore, beads were found to help counteract the negative effect of rPrP on amplification. It is tempting to speculate that the PMCAb format will help to improve the sensitivity of prion detection in body fluids such as blood or urine that might contain inhibitory compounds. Considering substantial enhancement in amplification yield, efficiency and robustness, PMCAb is a promising new platform for developing sensitive and rapid tests for prions, and producing PrP^Sc^ *in vitro* for structural studies.

The effect of beads on prion amplification can be explained by several mechanisms. Using amyloid fibrils produced from rPrP, we showed that sonication in the presence of beads effectively fragmented rPrP fibrils into pieces that were substantially smaller than those observed in the absence of beads (Fig. 9). This result suggests that beads might improve the efficiency of PrP^Sc^ fragmentation. Consistent with this mechanism, beads were found to enhance significantly the amplification efficiency of SSLOW PrP^Sc^, a strain which is deposited in the form of large plaques [25]. Sonication may not only fragment PrP^Sc^ particles but could also irreversibly damage or denature PrP^Sc^ and/or PrP^C^. We observed that during sonication, the beads rose from the bottom of the tubes and vibrated in the reaction mixtures. Perhaps, the presence of beads helps to redistribute the cavitation energy of bubbles into the much “softer” energy of mechanical vibration, making the conditions for breaking PrP^Sc^ particles more optimal.

In addition to more efficient fragmentation of PrP^Sc^ particles, the effect of beads could be attributed to a breakage of cellular debris and an increase in the accessibility of PrP^C^ and/or cellular cofactors essential for conversion. Considering that different strains or strains from different species might utilize a variety of cellular cofactors of different chemical natures [13], optimizing PMCA amplification might require a different bead material for some strains. Nevertheless, it is currently not known whether any of the proposed mechanisms provides an actual physical explanation for the effect of beads, which at this time should be considered empirical. While the mechanism of bead-induced effect remains to be elucidated in future studies, the PMCAb format offers immediate practical benefits.

In previous studies, only a small subfraction of PrP^C^ could be converted into PrP^Sc^ in PMCA, which raised concerns that only a fraction of PrP^C^ is susceptible to conversion. In an attempt to improve the conversion yield, an increase in the number of PMCA cycles [2] or the application of NBH from transgenic mice with high expression of endogenous PrP^C^ was employed [20], [21]. However, it is unclear whether these approaches can favorably change the balance between productive conversion and the competing reactions, which might include spontaneous oxidative modification of PrP^C^ [27], the self-cleavage of PrP^C^ [28] and unproductive misfolding. Increasing the time of a PMCA round or the concentration of a substrate is likely to impact both productive and unproductive pathways. The current work shows that an alternative approach that relies on a simple technical modification in the reaction format could be much more rewarding than biochemical approaches. An increase in the conversion yield suggested that beads selectively accelerate the rate of productive conversion of PrP^C^ into PrP^Sc^ without affecting competing reactions. Remarkably, a substantial fraction if not 100% of PrP^C^ could be converted into PrP^Sc^ in PMCAb (Fig. 1). This result argues that the amplification yield is not limited to a small subfraction of PrP^C^ susceptible to conversion or by cellular cofactors involved in the conversion reactions.

The most beneficial effect of beads on amplification was observed at high seed dilutions, i.e. at high PrP^C^/PrP^Sc^ ratios when the supply of PrP^C^ was unlimited (Fig. 3 and Fig. S4). In this case, beads improved the sensitivity of detection by at least 2 or 3 orders of magnitude. When seeded with high concentrations (10^3^ or 10^4^-dilution of scrapie brain material), the differences in amplification yield between PMCA and PMCAb was approximately 10-fold (Fig. 1). A 10-fold difference was also consistent with the difference in mean incubation time observed between the two groups after inoculation (Fig. 2). However, this difference must be considered tentative due to the low statistical significance of this measurement. Regardless, the bioassay confirmed that PMCAb amplifies prion infectivity at least equivalently to PMCA.

In our experience, prion amplification in PMCA is very sensitive to technical settings such as the precise position of a tube within the microplate horn, i.e. the distance of a tube from horn's surface and its center; the age of the sonicator's horn; the tube's shape. Furthermore, aging of sonicatior's horn and individual patterns of horn erosion with age cause time- and position-dependent variations in sonication power. As a result, it is difficult to obtain consistent amplification of PrP^Sc^ in experiments performed in different sonicators or even using the same sonicator as it ages. For instance, the differences in the yield of PrP^Sc^ amplification seen in lanes B6 and A1 in Fig. 1 were attributed to the aging of the sonicator's horn, as both these experiments were performed using the same sonicator but at a slightly different age. In our experience, Teflon beads significantly improve the robustness of PMCA making prion amplification less sensitive to technical variations, which are difficult to control. The new format should help to establish a PMCA-based approach for assays of prion infectivity.

## Methods

### Ethics statement

This study was carried out in strict accordance with the recommendations in the Guide for the Care and Use of Laboratory Animals of the National Institutes of Health. The protocol was approved by the Institutional Animal Care and Use Committee of the University of Maryland, Baltimore (Assurance Number A32000-01; Permit Number: 0309001).

### Protein misfolding cyclic amplification

Healthy hamsters were euthanized and immediately perfused with PBS, pH 7.4, supplemented with 5 mM EDTA. Brains were dissected, and 10% brain homogenate (w/v) was prepared using ice-cold conversion buffer and glass/Teflon tissue grinders cooled on ice and attached to a constant torque homogenizer (Heidolph RZR2020). The brains were ground at low speed until homogeneous, then 5 additional strokes completed the homogenization. The composition of conversion buffer was as previously described [29]: Ca^2+^-free and Mg^2+^-free PBS, pH 7.5, supplemented with 0.15 M NaCl, 1.0% Triton and 1 tablet of Complete protease inhibitors cocktail (Roche, Cat. # 1836145) per 50 ml of conversion buffer. The resulting 10% normal brain homogenate in conversion buffer was used as the substrate in PMCA reactions. To prepare seeds, 10% scrapie brain homogenates in PBS were serially diluted 10- to 10^14^-fold, as indicated, in the conversion buffer and 10 µl of the dilution were used to seed 90 µl of NBH in PMCA. Samples in 0.2 ml thin-wall PCR tubes (Fisher, Cat. # 14230205) were placed in a rack fixed inside Misonix S-3000 or S-4000 microplate horn, filled with 300 ml water. Two coils of rubber tubing attached to a circulating water bath were installed for maintaining 37°C inside the sonicator chamber. The standard sonication program consisted of 30 sec sonication pulses delivered at 50% to 70% efficiency applied every 30 min during a 24 hour period. For PMCA with beads, small (1.58 mm diameter) or large (2.38 mm diameter) Teflon beads (McMaster-Carr, Los Angeles, CA) were placed into the 0.2 ml tubes first using tweezers, then NBH and seeds were added. The following beads from Small Parts (www.smallparts.com) were tested in Figure 8: PTFE Ball Grade II (Teflon); Stainless Steel 440C Ball Grade 24; Neoprene Ball; Nylon Ball; EPDM Ball; Nitrile Rubber Ball; Stainless Steel 302 Ball Grade 100; Acetal Ball Grade I. The diameter of all beads was 2.38 mm except of Stainless Steel 440C Ball, which was 2 mm in diameter. The following low binding beads showed no effects on efficiency in PMCA: Silica Beads Low Binding 800 or 400 µm diameter, and Zirconium Beads Low Binding 200 or 100 µm diameter (all from OPS Diagnostics LLS, Lebanon, NJ).

In our experience, the amplification efficiency in PMCA depended strongly on the position of the tube within a microplate horn, i.e. distance of a tube from horn's surface to the tube and its center; and the age of the sonicator's horn. In the current studies, several Misonix sonicators were used, all equipped with horns less than one year old. The tubes were placed only in positions between 1.5 cm and 5 cm from the horn's center. Nevertheless, we experienced substantial variations in amplification efficiency in standard PMCA (no beads), which appear due to differences in the age of horns, individual patterns of horn corrosion or differences in the horizontal coordinates of tubes. In the presence of beads, the amplification was much more robust and showed only minor variations.

### Bioassay and estimation of the incubation times to disease

Weanling golden Syrian hamsters were inoculated intracerebrally with 50 µl each using the following inocula: animals of group 1 were inoculated with 263K brain homogenate diluted 10^4^-fold relative to whole brain in PBS, 1% BSA. For groups 2 and 3, 10 µl of 10% 263K scrapie brain homogenate were mixed with 90 µl of PBS and subjected to a sonication procedure equivalent to a single PMCA round (48 sonication cycles) in the absence of NBH. Sonication was performed either without beads (for group 2) or with 3 large beads (for group 3). Then, the sonication products were diluted 100-fold into PBS, 1% BSA to obtain final dilution of 10^−4^ relative to whole 263K brain for inoculation. For groups 4 and 5, PMCA reactions were seeded with 10^-4^-diluted 263K brain material, then six serial PMCA rounds were conducted in the absence of beads (for group 4) or presence of 3 large beads (for group 5) using 1∶10 dilutions between rounds. After the 6^th^ round of serial PMCA, the amplification products were diluted an additional 10-fold into PBS, 1% BSA to obtain final 10^10^-fold dilutions of the initial 263K brain material prior to inoculation.

In hamsters inoculated with the 263K scrapie strain, the asymptomatic period of infection lasts 60 to 160 days followed by a stereotypic clinical progression leading to death 2 to 3 weeks later. Individual symptoms, such as wobbling gait and head bobbing, are readily recognized but their onset is subtle and subject to large inter (and even intra) observer variability. Incubation time determinations are greatly improved by an empirical determination of endpoint [23]. The adult body weight of asymptomatic or uninfected hamsters is stable or increases slowly during adulthood but drops precipitously during symptomatic disease. Hamsters showing clear signs of early scrapie were individually caged and weighed daily. The weight history was plotted against time with a reference endpoint line marking 20% of the maximum weight registered. Animals were euthanized when their body weights dropped below 20% of maximum body weight and the incubation endpoint was taken as the time intercept of the 20% line.

### Proteinase K assay

To analyze production of PK-resistant PrP material in PMCA, 15 µl of each sample were supplemented with 2.5 µl SDS and 2.5 µl PK, to a final concentration of SDS and PK of 0.25% and 50 µg/ml respectively, followed by incubation at 37°C for 1 hour. The digestion was terminated by addition of SDS-sample buffer and boiling for 10 min. Samples were loaded onto NuPAGE 12% BisTris gels, transferred to PVDF membrane, and stained with 3F4 or D18 antibody for detecting hamster or mouse PrPs, respectively.

To analyze scrapie brain homogenates, an aliquot of 10% brain homogenate was mixed with an equal volume of 4% sarcosyl in PBS, supplemented with 50 mM Tris, pH 7.5, and digested with 20 µg/ml PK for 30 min at 37°C with 1000 rpm shaking (Eppendorf thermomixer). The reaction was stopped by adding 2 mM PMSF and SDS sample buffer. Samples were boiled for 10 min and loaded onto NuPAGE 12% BisTris gels. After transfer to PVDF membrane, PrP was detected with 3F4 antibody.

### Quantification of PrP^Sc^ by dot blotting

To obtain calibration curves for calculating of PMCA fold amplification, 10% brain homogenate from 263K animals was sonicated for 1 min and serially diluted into 10% NBH sonicated for 30 sec. PMCA samples as well as 263K dilutions were digested with 50 µg/ml PK for 1 h at 37°C. The reaction was stopped by addition of 2 mM PMSF. All samples were diluted 10-fold in PBS, and analyzed using a 96-well immunoassay similar to those previously published [30]. Our procedure employed the Bio-Dot microfiltration system (Bio-Rad, Hercules, CA) used according to the instruction manual. 50 µl of diluted samples were loaded into each well and allowed to bind to a 0.45 µm Trans-Blot nitrocellulose membrane (Bio-Rad, Hercules, CA). Following two washes with PBS, the membrane was removed, incubated for 30 min in 6 M GdnHCl to enable PrP denaturation, washed, and probed with 3F4 antibody according to the standard immunoblotting procedure. Chemiluminescent signal from the membrane was collected with a Typhoon 9200 Variable Mode Imager (Amersham Biosciences, Piscataway, NJ) and quantified with ImageQuant software (Amersham Biosciences).

### Atomic Force Microscopy imaging of rPrP-fibrils

Full-length hamster rPrP (residues 23-231, no tags) was expressed and purified as previously described [27], [28]. To prepare fibrils, the fibrillation reactions were conducted in 2M GdnHCl, 50 mM MES, pH 6.0 at 37°C at slow agitation (∼60 rpm) and rPrP concentration of 0.25 mg/ml [31]. 0.2 ml PCR tubes containing 100 µl solution of rPrP fibrils (10 µg/ml) dialyzed into 5 mM sodium acetate, pH 5.0, were placed in a rack fixed on the top of Misonix S-4000 microplate horn filled with 300 ml water and sonicated in the absence or presence of five small Teflon beads at 200 W ultrasound power for 30 s. Then, 5 µl of each sample was placed on a freshly cleaved piece of mica, incubated for 5 min, washed gently with Milli Q water, dried on air, and analyzed using a Pico LE AFM system (Agilent Technologies, Chandler, AZ) equipped with a PPP-NCH probe (Nanosensors, Switzerland) and operated in tapping mode. Topography/amplitude images (square size of 2 to 10 µm, 512×512 pixels) were obtained at 1 line/s scanning speed. Tip diameter was calibrated by obtaining images of 5-nm gold particles (BBinternational, UK) under the same scanning conditions. Images were analyzed with PicoScan software supplied with the instrument. Particle height was calculated directly from the topography profiles, while width and length were measured at the half-height and corrected for the tip diameter. Dimensions of 130–150 particles for each group from three independent experiments were measured.

## Supporting Information

Figure S1 Proteinase K assay of scrapie brain homogenates. Western blotting of scrapie brain homogenates from animal groups # 2, 3, 4 and 5. Two brain homogenates per group are shown. 10% brain homogenates were treated with 20 µg/ml PK for 30 min at 37C, 3F4 antibody was used for western blotting.
(0.16 MB TIF)

Figure S2 PMCAb does not produce PrP^Sc^ *de novo*. 10% NBH was subjected to a three rounds of serial PMCAb (with 3 large beads) in the absence of seeds and digested with PK. Each PMCA round consisted of 48 cycles; 10-fold dilutions were used for serial rounds. 32 independent reactions were analyzed. Undigested 10% NBH (lane 1) are showed as references.
(0.56 MB TIF)

Figure S3 Quantitative estimates of PrP^Sc^ amplification fold. (A) 263K scrapie brain material was serially diluted into 10% NBH to the final concentrations of scrapie brain ranging from 10% to 0.0625%, then digested with 50 µg/ml PK for 1 h at 37°C and analyzed using a 96-well dot blot. The signal intensity was measured using a Typhoon 9200 Variable Mode Imager and was found to be linear within the concentrations of scrapie brain homogenate from 0.0625% to 1% as shown in panel B. This concentration range was used to estimate the fold amplification of PrP^Sc^ in PMCA.
(0.33 MB TIF)

Figure S4 Analysis of PrP^Sc^ fold amplification in serial PMCA. 263K brain material was diluted 10^4^-fold into 10% NBH (lane 2) and subjected to a three rounds of PMCA in the absence of beads (top panel) or presence of 3 large beads (bottom panel) and digested with PK. The material amplified in each round was diluted 10-, 20-, 100-, or 1000-fold into 10% NBH for the next PMCA round, as indicated. Each PMCA round consisted of 48 cycles. Undigested 10% NBH (lane 1) is provided as a reference.
(0.44 MB TIF)
