# Supplementary figures and images for: Highly Efficient Protein Misfolding Cyclic Amplification
Source: PLoS Pathog. 2011 Feb 10;7(2):e1001277. doi: 10.1371/journal.ppat.1001277 (PMC3037363; doi:10.1371/journal.ppat.1001277)

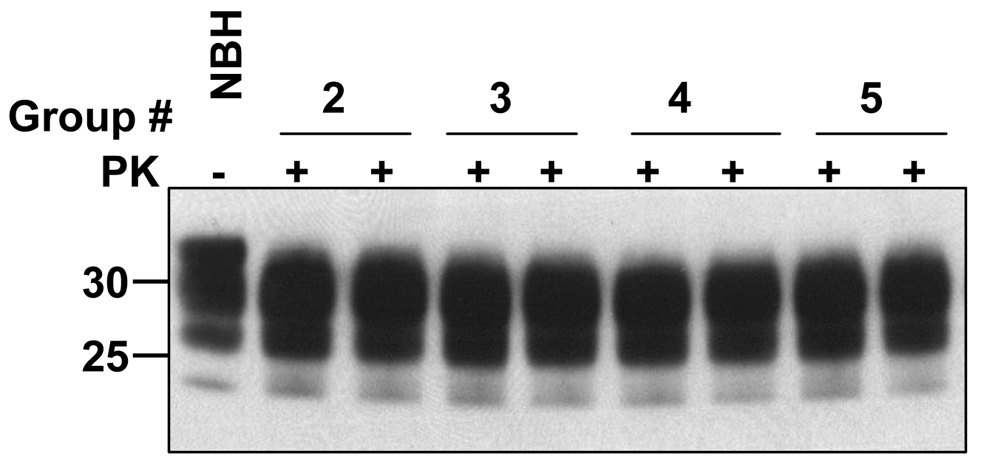

Supplement: Figure S1 — Proteinase K assay of scrapie brain homogenates. Western blotting of scrapie brain homogenates from animal groups # 2, 3, 4 and 5. Two brain homogenates per group are shown. 10% brain homogenates were treated with 20 µg/ml PK for 30 min at 37C, 3F4 antibody was used for western blotting. (0.16 MB TIF) [file ppat.1001277.s001.tif]

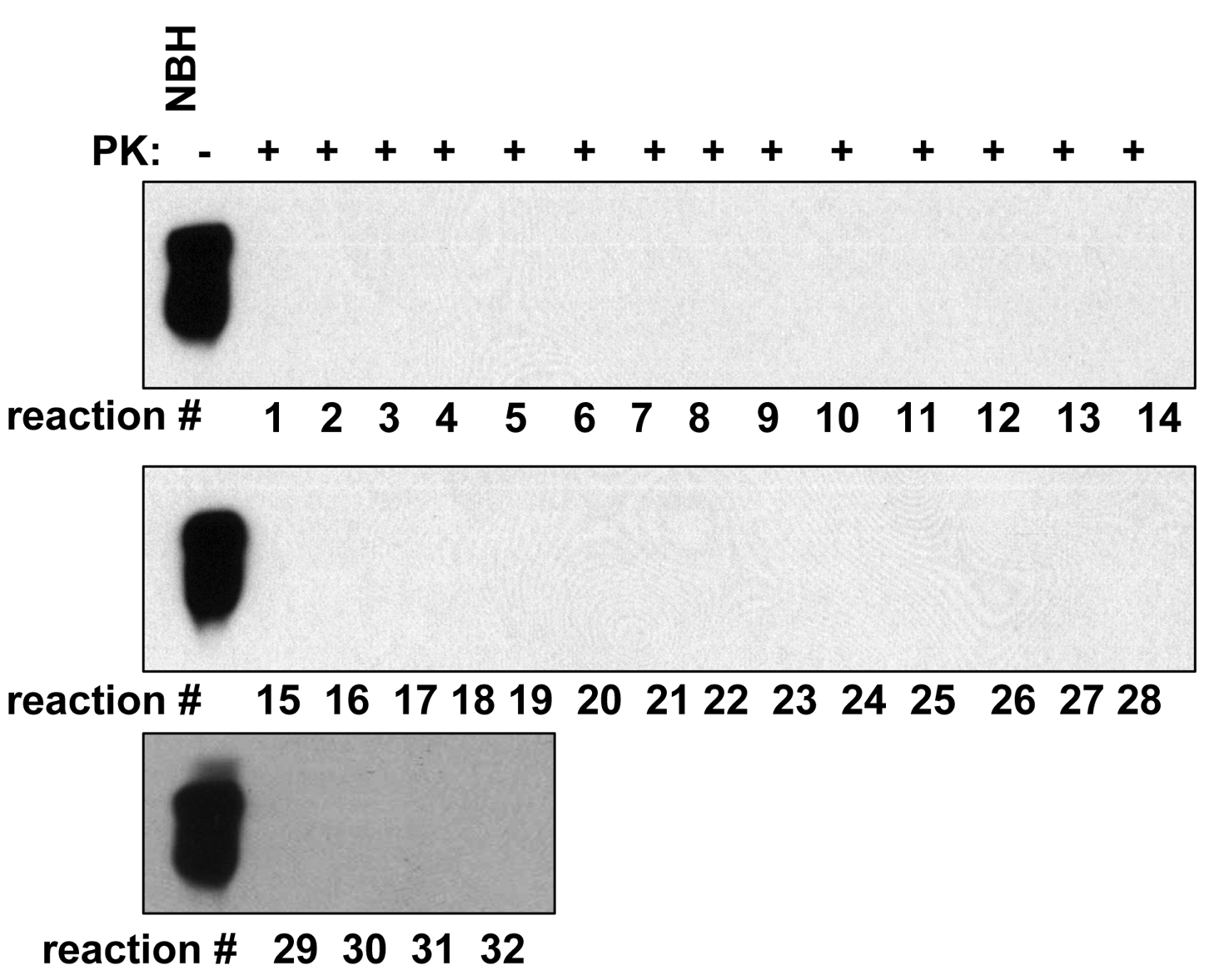

Supplement: Figure S2 — PMCAb does not produce PrPSc de novo. 10% NBH was subjected to a three rounds of serial PMCAb (with 3 large beads) in the absence of seeds and digested with PK. Each PMCA round consisted of 48 cycles; 10-fold dilutions were used for serial rounds. 32 independent reactions were analyzed. Undigested 10% NBH (lane 1) are showed as references. (0.56 MB TIF) [file ppat.1001277.s002.tif]

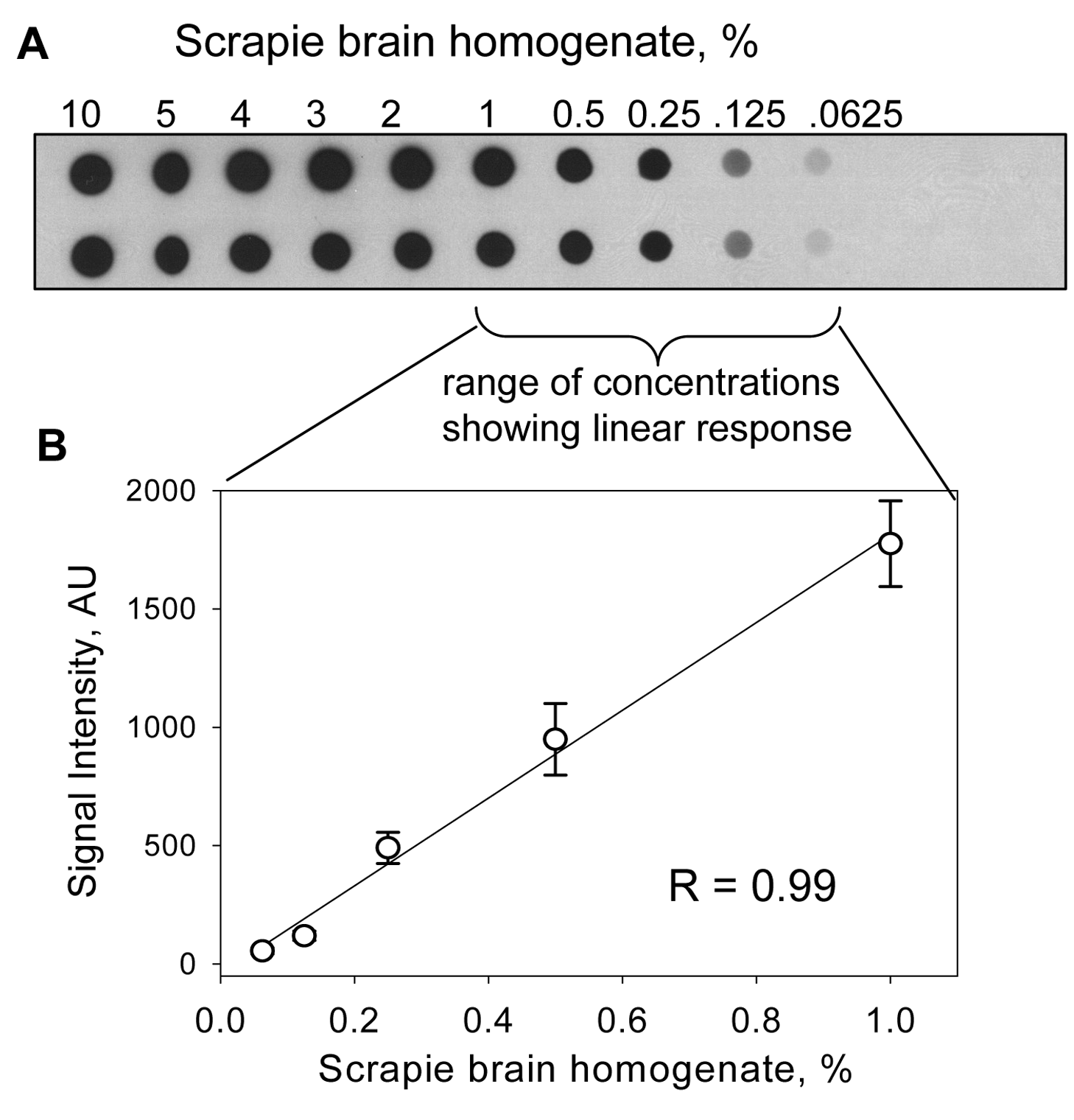

Supplement: Figure S3 — Quantitative estimates of PrPSc amplification fold. (A) 263K scrapie brain material was serially diluted into 10% NBH to the final concentrations of scrapie brain ranging from 10% to 0.0625%, then digested with 50 µg/ml PK for 1 h at 37°C and analyzed using a 96-well dot blot. The signal intensity was measured using a Typhoon 9200 Variable Mode Imager and was found to be linear within the concentrations of scrapie brain homogenate from 0.0625% to 1% as shown in panel B. This concentration range was used to estimate the fold amplification of PrPSc in PMCA. (0.33 MB TIF) [file ppat.1001277.s003.tif]

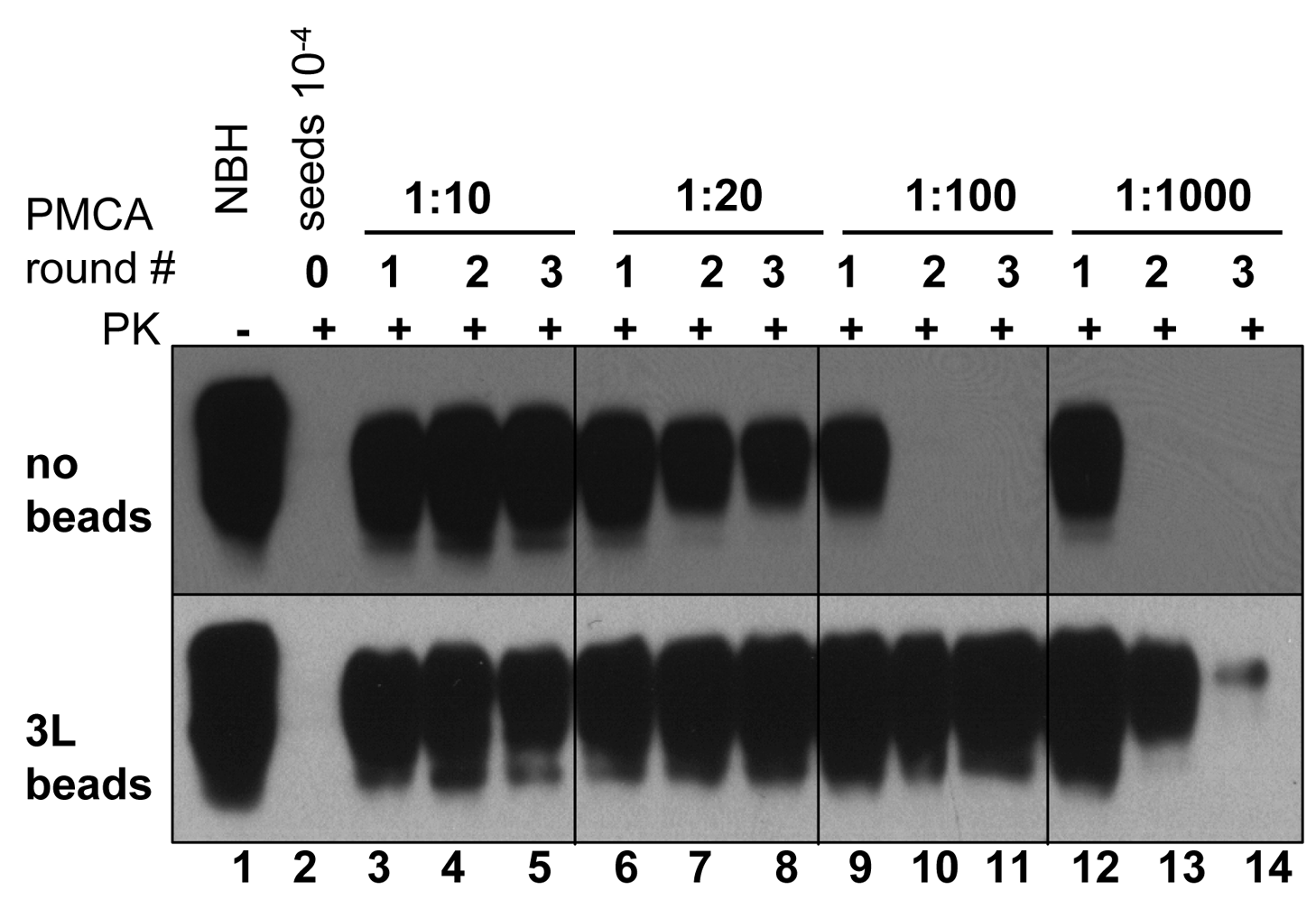

Supplement: Figure S4 — Analysis of PrPSc fold amplification in serial PMCA. 263K brain material was diluted 104-fold into 10% NBH (lane 2) and subjected to a three rounds of PMCA in the absence of beads (top panel) or presence of 3 large beads (bottom panel) and digested with PK. The material amplified in each round was diluted 10-, 20-, 100-, or 1000-fold into 10% NBH for the next PMCA round, as indicated. Each PMCA round consisted of 48 cycles. Undigested 10% NBH (lane 1) is provided as a reference. (0.44 MB TIF) [file ppat.1001277.s004.tif]
